# Experimental model for the study of retrograde flow

**DOI:** 10.1590/1677-5449.008915

**Published:** 2016

**Authors:** Cesar Roberto Busato, Carlos Alberto Lima Utrabo, Leandro Cavalcante Lipinski, Mario Rodrigues Montemór, Eduardo de Souza Tolentino, Fernanda Aparecida de Oliveira Busato Nascimento, Marcio Dias Guilherme

**Affiliations:** 1 Universidade Estadual de Ponta Grossa – UEPG, Departamento de Medicina, Ponta Grossa, PR, Brazil.

**Keywords:** ischemia, arteriovenous fistula, microcirculation, isquemia, fístula arteriovenosa, microcirculação

## Abstract

**Background:**

Venous arterialization has been adopted as a strategy for salvage of limbs in critical ischemia without the distal arterial bed, with successful outcomes, but the mechanisms by which irrigation of the extremities takes place are still unknown.

**Objectives:**

To develop an experimental model to test hypotheses that could explain the mechanisms of blood supply in venous arterialization.

**Methods:**

Eleven pigs underwent a period of hind limb ischemia followed by reperfusion achieved by venous arterialization, after interposition of conduits filled with 10 ml (5 animals – group 1) or 1 ml (6 animals – group 2) of China Ink. After euthanasia, the limbs were amputated and underwent histological analysis.

**Results:**

Under optical microscopy, ink staining was observed in the arteriolar lumen of six (55%) of the eleven pigs used in the experiment; four (80%) out of five from group 1 and two (33%) out of six from group 2.

**Conclusions:**

The experimental model was capable of testing the hypothesis. The presence of China Ink in the arteriolar lumen shows that it is possible to supply the arterial vessels by means of venous arterialization.

## Resumo

**Contexto:**

A arterialização venosa tem sido adotada com bons resultados como estratégia para salvar membros em isquemia crítica sem leito arterial distal. No entanto, os mecanismos pelos quais a irrigação das extremidades ocorre permanecem desconhecidos.

**Objetivos:**

Desenvolver um modelo experimental para testar hipóteses que podem explicar os mecanismos de nutrição em arterialização venosa.

**Métodos:**

Onze porcos foram submetidos a um período de isquemia seguida de reperfusão do membro posterior, realizada por arterialização venosa, com interposição de condutos preenchidos com 10 mL (cinco animais - grupo 1) e 1 mL (seis animais - grupo 2) de tinta da China. Após a eutanásia, os membros foram amputados e submetidos a análise histológica.

**Resultados:**

Na microscopia óptica, o pigmento foi encontrado no lúmen de arteríolas de seis (55%) dos 11 porcos utilizados no experimento; quatro (80%) de cinco animais eram do grupo 1 e dois (33%) de seis animais eram do grupo 2.

**Conclusões:**

O modelo experimental utilizado foi capaz de testar a hipótese. A presença de tinta da China no lúmen arteriolar mostra que é possível alcançar o vaso arterial por meio de arterialização venosa.

## INTRODUCTION

Each year 1,000 new cases of critical limb ischemia (CLI) are identified in Europe and North America per population of one million inhabitants.^1^ Studies suggest that in developed countries about 25% of CLI patients undergo primary amputation, while in developing countries, where there is a lack of specialized programs to salvage the limb, amputation has been adopted as a first-line therapy.^2^

Great strides taken in development of techniques and devices have offered researchers the opportunity to disseminate endovascular revascularization procedures as a means to restore blood flow in CLI.^2^ In acute ischemia, without the distal arterial bed, it is not possible to divert arterial blood to the extremity, but it may be possible to supply blood to the structure involved by diverting flow through venous circulation in the retrograde direction. This technique is based on the theory that in the absence of primary arterial pressure in the arterioles, blood supplied through the venous system by means of arterial pressure is able to feed peripheral tissues and supply adequate oxygenation.^3^^,^ 4 Venous arterialization has been adopted as a strategy for limb salvage in acute ischemia without the distal arterial bed. Studies^3^^-^ 14 have reported successful outcomes, all evidence-based. Nevertheless, the mechanisms through which irrigation of the extremities takes place are still unknown.

The authors of this study decided to attempt to develop an experimental model which would enable them to reproduce the scenarios of ischemia and subsequent reperfusion to allow them to test the hypothesis that venous arterialization induces retrograde flow from the higher-pressure venules (proximal arterial extremity) to the lower-pressure arterioles.

## METHODS

This research project was approved by the Animal Research Ethics Committee (CEUA 009/13) and conducted at the Universidade Estadual de Ponta Grossa (UEPG), Ponta Grossa, PR, Brazil, during the first semester of 2014. Eleven male and female Landrace Large White Cross pigs, all from the same litter and with a mean weight of 20 kg, were allocated to two groups, five in group 1 and six in group 2. The pigs were pre-anesthetized with 0.4 mg/kg of acepromazine, 0.2 mg/kg of xylazine and 14 mg/kg of ketamine. Anesthesia was induced with propofol (5 mg/kg) and then the pigs were subjected to tracheal intubation. The animals were kept on inhalation anesthesia with isoflurane via mechanical ventilation with an oxygen volume of 10 ml/kg.

These animals had originally been made available to students at the Medical School for use in practical classes in Operating Technique. At the end of these procedures those that were in good clinical conditions were used for the procedure described here, before being euthanized,.

**Figure 1:**
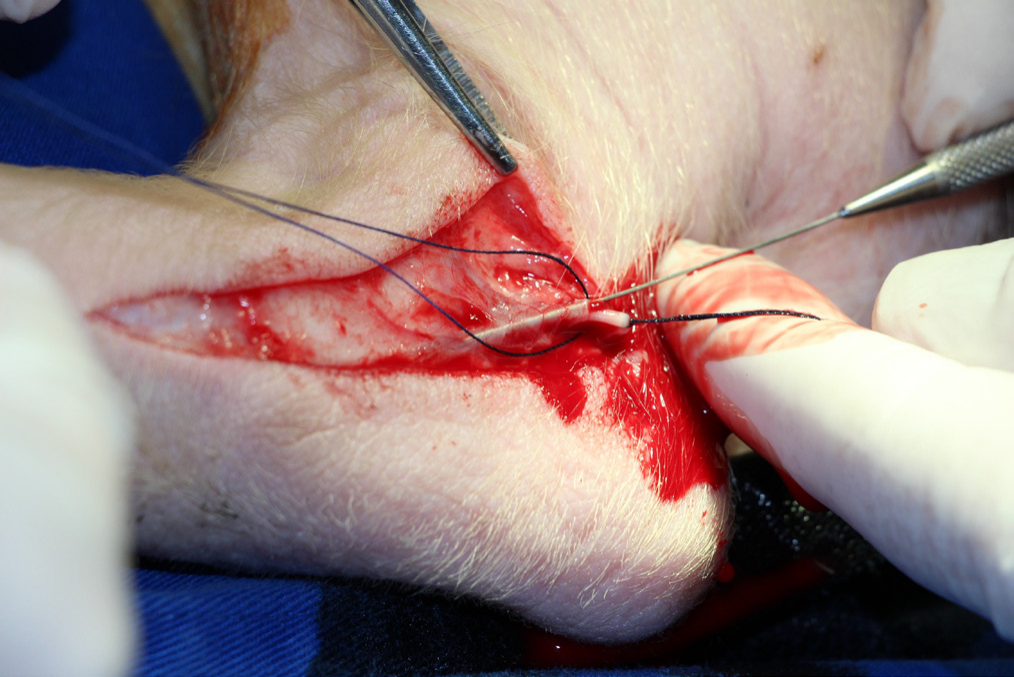
Dissection, ligature and valve disruption of the small saphenous vein.

The porcine model has a similar vascular anatomy to humans. Initially, the small saphenous vein was dissected and systemic anticoagulation was achieved with 5000 U of intravenous heparin, followed by proximal ligature and downstream rupture of the venous valves with a Lengua valvulotome (Figure 1). Distal canalization was performed using a number 4 probe.

**Figure 2:**
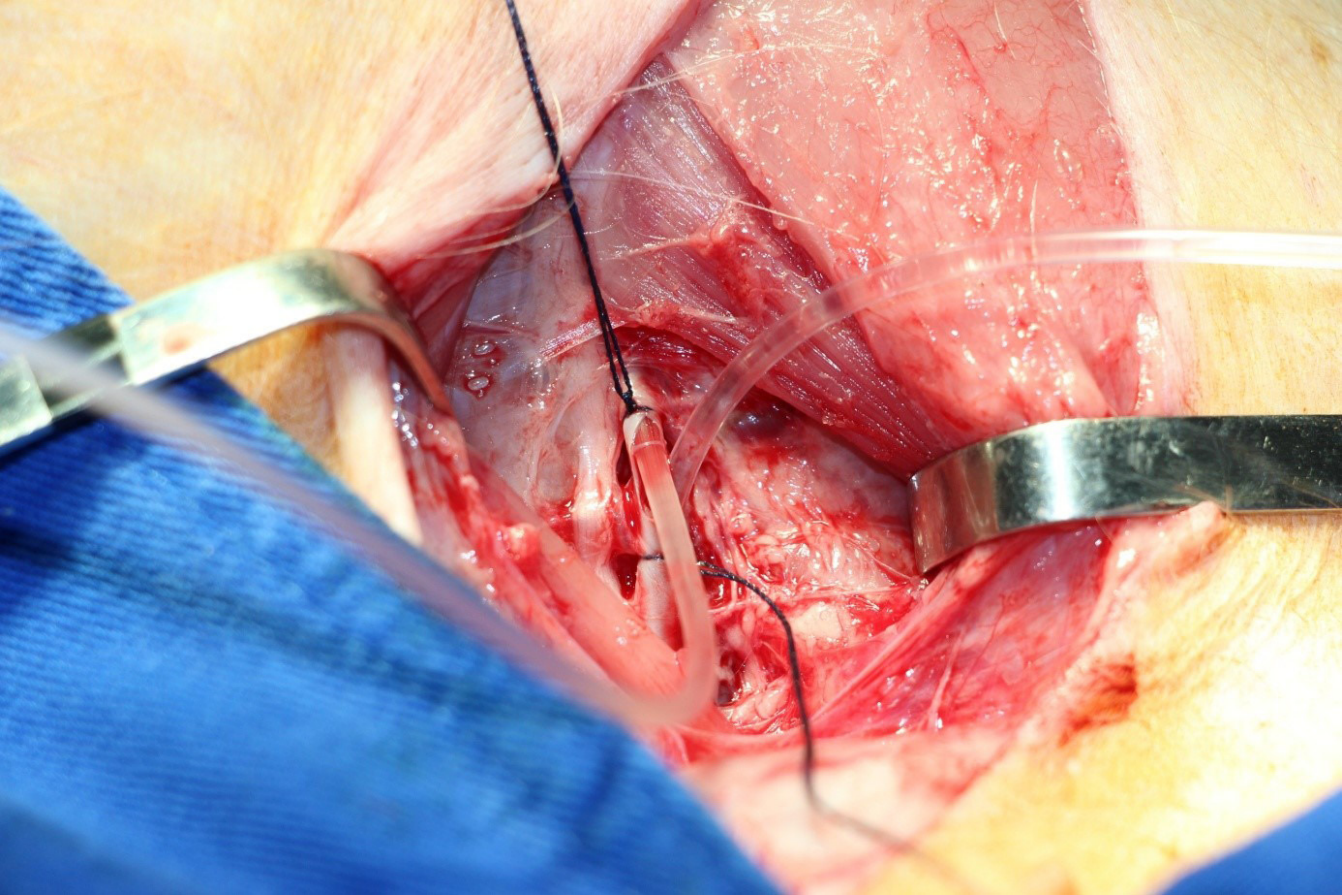
Dissection, section, and proximal and distal canalization of the common femoral artery.

The common femoral artery and its superficial and deep branches were then dissected. The proximal (common femoral artery) and distal (superficial femoral artery) ends were canalized using a number 6 probe, so that the deep femoral artery was excluded, and a period of ischemia lasting approximately 30 minutes was initiated (Figure 2).

The proximal extremity of the femoral artery was then connected to the small saphenous vein, and reperfusion was allowed to progress for 30 minutes.

**Figure 3:**
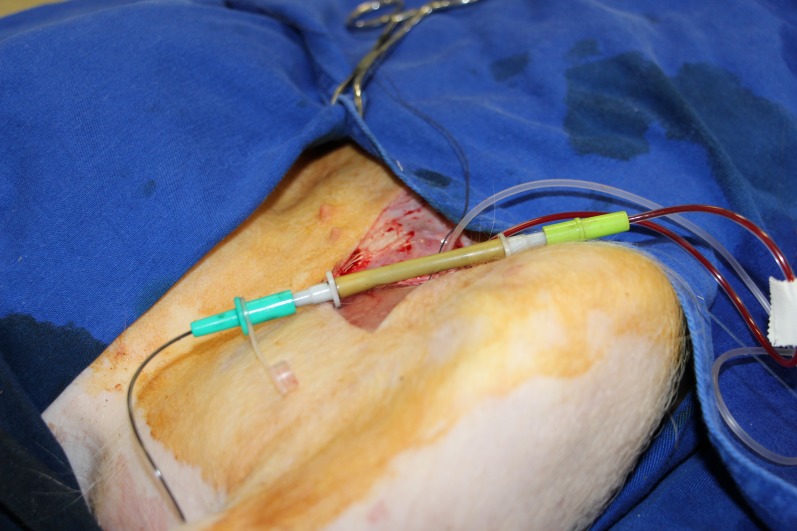
Interposition of China Ink between the proximal arterial extremity and the distal venous extremity.

After this period had elapsed, a conduit interposition was performed with 1/10 China Ink and saline solution, between the proximal arterial and distal venous extremities, until all the dye had been transferred to the venous end (Figure 3), and kept for additional 30 minutes. The five animals in group 1 and six in group 2 were administered 10 ml and 1 ml of the ink solution respectively.

At the end of the procedure, the animals were euthanized in accordance with Federal Board of Veterinary Medicine (CFMV) resolution 1000/2012. Amputation at was performed at the level of the calcaneus. The amputated limb was preserved in 10% formaldehyde for histological analysis. Slides were prepared with hematoxylin-eosin staining and inspected for the presence of China Ink in arterioles.

## RESULTS

**Figure 4:**
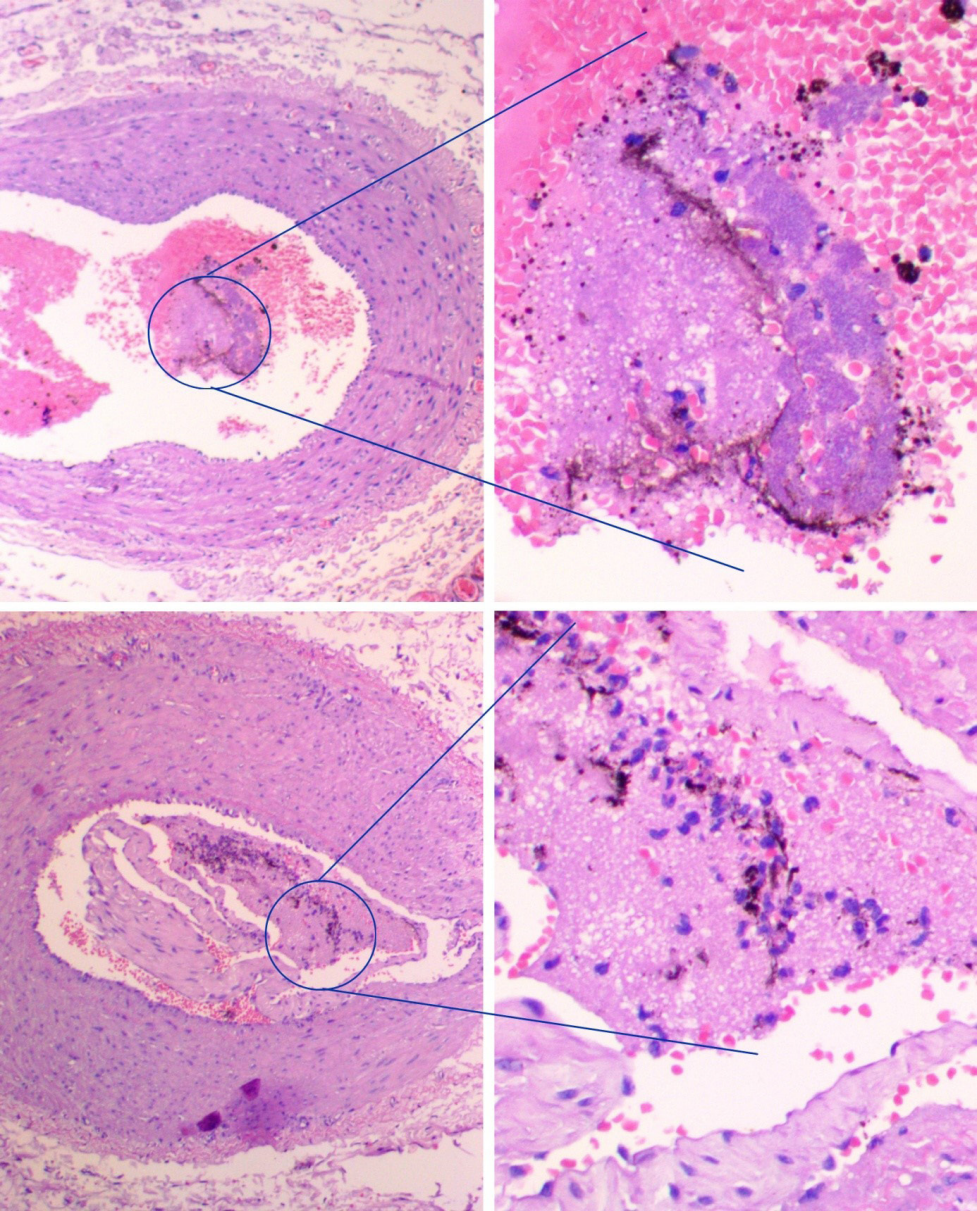
Presence of China Ink stain in the arterioles of pigs subjected to venous arterialization.

Histological analysis detected presence of the stain in the arterioles of six (55%) out of the eleven pigs used in the experiment (Figure 4), four (80%) out of five from group 1 and two (33%) out of six from group 2.

The quantity of stain found by the observer was classified in a subjective manner, based on the greatest quantity, from + to ++++, as shown in Table 1. Microscopy did not detect any areas of ischemic tissue.

**Table 1:** Presence and quantities of China Ink found in histological analysis of pig limbs subjected to retrograde arterialization.

**Animal identification**	**Group**	**Presence of ink staining**	**Quantity**
Pig 1	1	Yes	++++/++++
Pig 2	1	Yes	++/++++
Pig 3	2	No	-
Pig 4	2	No	-
Pig 5	2	Yes	++/++++
Pig 6	2	Yes	+/++++
Pig 7	2	No	-
Pig 8	1	Yes	+/++++
Pig 9	1	Yes	+/++++
Pig 10	1	No	-
Pig 11	2	No	-

## DISCUSSION

Attempts to supply arterial blood to territories suffering from major ischemia via a retrograde venous approach date from the early 20th century. Observations that a much greater force than provided by normal blood pressure is required to overcome the obstacles created by venous valve also date from the same period. This method was condemned by the poor outcomes resulting from creation of arteriovenous fistulas in femoral vessels.^6^

In 1948, Kunlin, having performed the first successful bypass surgery, started a new era in the treatment of ischemia. Improvements in suture materials and angiographic techniques and the adoption of microsurgery have made it possible to implant bypasses in small arteries at the level of the ankle and foot. Lengua returned to this old idea, but changed its method of application, achieving arterialization of foot veins using a bypass. This technique was first used in 1974 in three diabetic patients with promising results, but it was not well received and it was only 20 years later that it began to be adopted because of publications about its successful use by other vascular surgery teams.^9^

More recently, other medical, surgical, and endovascular advances have been used. However, all over the world, amputations continue to be performed of lower limbs with critical ischemia and no distal arterial run-off, mainly in diabetic patients.^6^^,^ 9

According to Taylor, distal venous arterialization is a unique procedure that has exciting possibilities for limb salvage and merits further investigation. Increased use of this procedure could help to reduce the number of limbs amputated because of vascular disease.^4^^,^ 6

Conditions that justify indicating this procedure are atherosclerosis obliterans, especially when associated with diabetes mellitus, thromboangiitis obliterans in most cases and popliteal artery aneurysms with distal bed thrombosis.^3^^,^ 8

Inverted venous graft,^9^^,^ 12 great saphenous vein in situ^3^ and endoluminal selective deep calf venous arterialization oriented by an angiosome model^5^ have all been used. The results depend more on the characteristics of the patient than on the technique itself. However, success rates are limited.^13^

Publications describing successful use include a prospective randomized study that compared distal venous arterializations with conservative treatment using antiplatelet drugs.^7^

A case-control study evaluated the clinical outcomes in 40 patients with critical limb ischemia, 21 treated with distal venous arterializations and 19 with conventional surgery to perform pedal bypass. In the distal venous arterialization group, early occlusion was 15%, 1-year patency was 71%, and limb salvage was 53%. In the pedal bypass group, early occlusion was 23%, one-year patency was 75% and limb salvage was 47%. Limb salvage after distal venous arterialization was equal to limb salvage after pedal bypass surgery.^14^

A meta-analysis selected 56 studies for comprehensive review. Seven patient series, comprising 228 patients, matched the selection criteria. Overall, 1-year foot preservation was 71% (95% CI: 64-77%) and 1-year secondary patency was 46% (95% CI: 39-53%). The large majority of patients in whom major amputation was avoided experienced successful wound healing, disappearance of pain at rest and absence of serious complications.^10^

After 24 months’ follow-up, Mutiranguara observed a survival rate of 87.5%, and limb salvage and graft patency rates of 76.02 and 49.17% respectively.^11^

Arterialization of the foot is possible, effective and long-lasting, probably thanks to induction of neo-arteriogenesis and neo-angiogenesis that maintain the benefits even after the bypass has occluded (temporary function).^9^ This mechanism is based on the assumption that there should be a positive pressure gradient between the arterialized venous blood and arterial network which supply adequate oxygenation until these mechanisms can meet.^3^^,^ 4

All observations were evidence-based and the mechanisms through which the irrigation of the extremities takes place are still unknown.

Sasajima,^15^^,^ 16 Ozbeck,^17^ Koyama,^18^ and Lengua^19^^,^ 20 have conducted experimental studies attempting to show the mechanisms through which the successful clinical results are obtained. According to Sasajima,^16^ hypoxia stimulates endothelial growth factor, thereby inducing angiogenesis of the arterialized tissue. Arteriographic studies support these findings. According to Ozbeck,^17^ the surgery induces an inflammatory process that heals incisions and activates angiogenesis.

Koyama^18^ suggests that retrograde peripheral blood flow is rapidly established after distal venous arterialization surgery. There is as yet no rational explanation for this phenomenon. He speculates that the remarkably thin venular walls are capable of considerable and rapid distension when subjected to increased haemostatic pressure. The increase in venule diameter in response to increased blood pressure renders their valve leaflets incompetent, so that the valves can no longer close the vessel lumen.

Lengua^19^ presents hypotheses for the physiology of arterialization. The poor peripheral blood pressure resulting from the significant arterial occlusion in the extremity is a factor that facilitates the relatively high-pressure back flow of the arterialized venous blood, similar to Koyama’s suggestion. Using arteriography, Lengua^20^ observed that once the fistula was created there was a great increase in microcirculation, thereby corroborating the findings of Ozbeck and Sasajima.

The animal model described here offers an anatomy similar to the human, although the internal iliac artery has a posterior direction and the external is lower. The common, superficial and deep femoral arteries are exposed and by channeling the common and superficial femoral arteries, the deep femoral artery is excluded from the circuit.

By demonstrating the presence of China Ink in the arterioles, our research has revealed that there is retrograde flow from the venules (from the proximal arterial extremity) to the arterioles due to arterialization. It is possible that the pressure gradient created by ligation of the common femoral artery and arterialization of the venous extremity allows the arterial blood to flow through the venous bed. Demonstration of this phenomenon with pigment in all of the animals tested would be dependent on variables such as using an adequate volume of pigment and the time elapsed ink injection and limb amputation. Venous drainage of the pigment is likely to be directly proportional to the elapsed time and inversely proportional to the perfusion volume.

Although the model produced acute limb ischemia, clinical results with chronic patients suggests that in both situations venous arterialization is governed by hydrodynamics laws.

This model can be used for comparison of arterialized extremities with extremities in other states, such as ischemia and normal circulation, using direct parameters, such as appearance, temperature measures and pulsed wave Doppler and indirect parameters such as blood gases, lactate and creatine phosphokinase.

We believe that further experiments employing longer duration ischemia and arterialization could be conducted to test other hypotheses and variables.

The experimental model reproduced scenarios of ischemia and reperfusion by means of the venous arterialization and was capable of testing the hypothesis formulated. The presence of China Ink in the arterioles shows that it is possible to supply them by means of venous arterialization.
